# Simulation and Evaluation of Pollution Load Reduction Scenarios for Water Environmental Management: A Case Study of Inflow River of Taihu Lake, China

**DOI:** 10.3390/ijerph110909306

**Published:** 2014-09-09

**Authors:** Ruibin Zhang, Xin Qian, Wenting Zhu, Hailong Gao, Wei Hu, Jinhua Wang

**Affiliations:** State Key Laboratory of Pollution Control and Resource Reuse, School of the Environment, Nanjing University, Nanjing 210046, China; E-Mails: zhangrb88@126.com (R.Z.); dtzwt1987@126.com (W.Z.); njughl@gmail.com (H.G.); huw913@gmail.com (W.H.); huawangjin@163.com (J.W.)

**Keywords:** water quality modeling, pollution load reduction scenario, QUAL2K model, simulation, evaluation, Wujin River, water environmental management

## Abstract

In the beginning of the 21st century, the deterioration of water quality in Taihu Lake, China, has caused widespread concern. The primary source of pollution in Taihu Lake is river inflows. Effective pollution load reduction scenarios need to be implemented in these rivers in order to improve the water quality of Taihu Lake. It is important to select appropriate pollution load reduction scenarios for achieving particular goals. The aim of this study was to facilitate the selection of appropriate scenarios. The QUAL2K model for river water quality was used to simulate the effects of a range of pollution load reduction scenarios in the Wujin River, which is one of the major inflow rivers of Taihu Lake. The model was calibrated for the year 2010 and validated for the year 2011. Various pollution load reduction scenarios were assessed using an analytic hierarchy process, and increasing rates of evaluation indicators were predicted using the Delphi method. The results showed that control of pollution from the source is the optimal method for pollution prevention and control, and the method of “Treatment after Pollution” has bad environmental, social and ecological effects. The method applied in this study can assist for environmental managers to select suitable pollution load reduction scenarios for achieving various objectives.

## 1. Introduction

The pollution load of Taihu Lake Basin, China is primarily from river inflows. Hence, the key to protecting the water environment of the lake is interception of pollutants in these river inflows. In recent decades, rivers have become the main recipients of wastewater, brought about by a developing economy and growing population [1,2,3]. Pollution of rivers is one of the most prominent water environmental problems, and is closely related to economic development and quality of life in the region [4,5,6,7]. To solve these environmental problems, managers must select appropriate pollution load reduction scenarios for achieving particular goals, thus decisions between alternative scenarios can be a difficult task for water environmental managers.

There have been many studies on water treatment technologies and pollution prevention scenarios; however, there have been few studies on the quantitative evaluation of pollution control scenarios, especially the optimization and selection of appropriate water pollution control schemes for meeting different objectives [8,9,10,11,12]. The goal of this study was to select appropriate pollution load reduction scenarios for particular objectives using a simulation method. The Wujin River was selected because it is one of the major inflow rivers of Taihu Lake.

In the last two decades, many water quality models have been developed for various types of water bodies. For example, Hamilton and Schladow [13] applied a DYRESM model to analyze spatial changes in water quality. Wang and Cresser [14] used a QUESTS1D model to evaluate alternative river management options in a tidal river. QUAL2E was applied in studies by Drolc and Koncan [15], Ning *et al.* [16], and Palmieri and de Carvalho [17]. Pelletier *et al.* [18] validated the flexibility and applicability of the QUAL2K model for simulation of river water quality. Some useful applications of QUAL2K have also been published [3,19,20,21,22]. QUAL2K was selected for this study because of its popularity and ease of application.

The objective of this study was to select appropriate pollution load reduction scenarios for particular goals. First, the QUAL2K model was applied to calibrate and validate parameters of Wujin River. Second, a variety of pollution load reduction scenarios were simulated using the calibrated QUAL2K model. Third, the pollution load reduction rates of different scenarios, as required for meeting water quality standards, were calculated for Wujin River water entering Taihu Lake. Finally, various pollution load reduction scenarios were assessed using an analytic hierarchy process to comprehensively evaluate their effects of environmental, social, economic, ecological, and costs.

## 2. Material and Methods

### 2.1. Study Area

The Wujin River, situated in the north of Taihu Lake Basin, was selected as the study area. The mainstream of the river is 35.0 km long, about 2–3 m deep and 25–30 m wide. It is the main river of Changzhou City, and flows into Meiliang Bay and Zhushan Bay of Taihu Lake. Both Meiliang Bay and Zhushan Bay are important sources of drinking water and are important tourist destinations in Taihu Lake; however, Meiliang Bay and Zhushan Bay are the most seriously affected by cyanobacteria blooms [23,24]. The water quality of Wujin River is generally inferior to the Grade V water quality standards of China [25].

**Figure 1:**
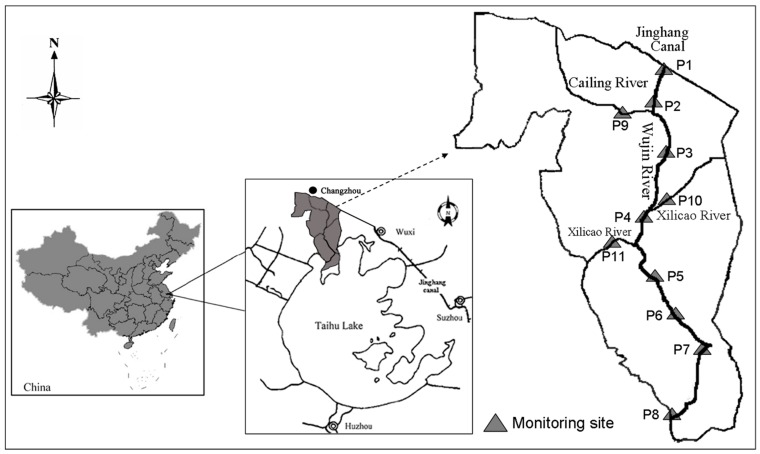
Study area and monitoring sites along the Wujin River.

The study area included 35.0 km of the Wujin River, with a watershed of 423.6 km^2^ (Figure 1). The river is an important water source for drinking, irrigation, industry and entertainment for nearly 450,000 people. About 30 years ago, the water from the Wujin River was potable; however, rapid socioeconomic development in the area has led to increased emissions of untreated wastewater and pollutants from domestic, industrial and agricultural processes, which has resulted in decreased water quality in the river. Sites P1–P8 along the Wujin River and P9–P11 along the tributaries were selected as monitoring sites (Figure 1). The latitude and longitude of monitoring points are shown in Table 1.

**Table 1:** The latitude and longitude of monitoring points.

Monitoring Point	P1	P2	P3	P4	P5	P6	P7	P8	P9	P10	P11
Latitude (N)	31°42′45″	31°41'18"	31°39'2"	31°36'15"	31°33'12"	31°31'44"	31°30'27"	31°27'49"	31°40'53"	31°36'20"	31°35'6"
Longitude (E)	120°4′22″	120°3'52"	120°4'35"	120°3'36"	120°3'55"	120°4'53"	120°6'13"	120°5'25"	120°1'58"	120°3'51"	120°2'11"

### 2.2. QUAL2K Model

QUAL2K is a one-dimensional river and stream water quality model that is an upgraded version of the QUAL2E model [26]. The QUAL2K model, which was developed by the US Environmental Protection Agency, considers the stream as a one-dimensional channel with steady flow that is non-uniform and considers the influence of point source and non-point source pollution loads [27].

QUAL2K can simulate the migration and transformation of a wide variety of constituents including dissolved oxygen (DO), temperature, biochemical oxygen demand (BOD), chemical oxygen demand (COD), organic nitrogen, ammonia nitrogen (NH_3_-N), nitrate nitrogen (NO_3_-N), total nitrogen (TN), sediment oxygen demand (SOD), organic phosphorus, inorganic phosphorus, total phosphorus (TP), phytoplankton and algae. The illustrations and uses of this model are described in detail in the QUAL2K user’s manual [27]. The degradation coefficient of various pollutants and hydrodynamic parameters are required for simulation of the water quality.

### 2.3. Simulation Method of Load Reduction Scenarios

#### 2.3.1. Input Data

**Figure 2:**
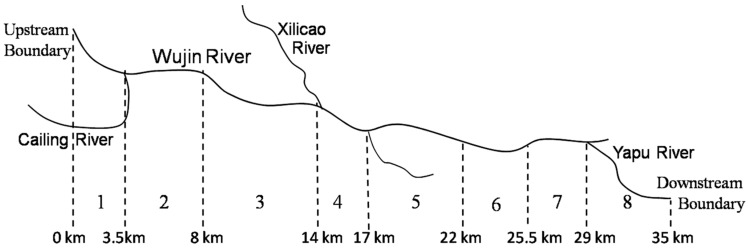
System segmentation and three tributaries of Wujin River.

The 35.0 km length of Wujin River was divided into eight reaches. There were 70 computational elements with a length of 500 m. In the headwater of the river, average annual flow is 2.63 m^3^/s, average annual temperature is 18.7 °C, and flow velocities are in the range of 0.04–0.07 m/s. Figure 2 shows the system segmentation and three tributaries of Wujin River. Table 2 shows the locations and hydraulic characteristics of eight reaches including manning coefficient, bottom algae coverage and bottom SOD coverage and so on. The locations and water qualities of main tributaries are shown in Table 3.

**Table 2:** The locations and hydraulic characteristics of eight reaches.

Reach	Length (km)	Location (km)	Latitude	Longitude	Bottom Width (m)	Manning Coefficient	Bottom Algae Coverage (%)	Bottom SOD Coverage (%)
1	3.5	3.5	31°41'18"N	120°3'52"E	25	0.03	50	100
2	4.5	8.0	31°39'2"N	120°4'35"E	25	0.03	50	100
3	6.0	14.0	31°36'15"N	120°3'36"E	25	0.03	50	100
4	3.0	17.0	31°35'9"N	120°3'7"E	25	0.04	70	100
5	5.0	22.0	31°33'12"N	120°3'55"E	30	0.04	70	100
6	3.5	25.5	31°31'44"N	120°4'53"E	30	0.05	80	100
7	3.5	29.0	31°30'27"N	120°6'13"E	30	0.05	100	100
8	6.0	35.0	31°27'49"N	120°5'25"E	30	0.05	100	100

**Table 3:** The locations and water qualities of main tributaries.

Tributary	Location (km)	Monitoring Sites	Flow (m^3^/s)	COD (mg/L)	NH_3_-N (mg/L)	TN (mg/L)	TP (mg/L)
Cailing River	3.5	P9	1.43	44.1	3.06	7.27	0.35
Xilicao River upstream	14.0	P10	2.81	31.2	1.75	4.16	0.25
Xilicao River downstream	17.0	P11	3.07	34.3	2.23	5.30	0.23

There are 10 point source inputs to the Wujin River: seven main point sources in the Wujin River watershed and three main tributaries. There are nine non-point sources as determined by different inflow concentrations. The flow and concentration of pollution sources are shown in Table 4 and Table 5. The concentration coming from pollution sources were obtained by the ratio of annual pollutants emissions and wastewater emissions of each pollution source. The data of annual pollutants emissions and wastewater emissions were obtained from data collection and field survey.

The input parameters involved in QUAL2K were temperature, flow, COD, DO, organic nitrogen, NH_3_-N, NO_3_-N, inorganic phosphorus and organic phosphorus. The level of phytoplankton in the Wujin River is negligible. The water qualities at uppermost station P1 was considered as upstream boundary. The downstream boundary was not prescribed for it has no effect in modeling.

**Table 4:** Flow and concentration of main point sources.

Location (km)	Flow (m^3^/d)	COD (mg/L)	NH_3_-N (mg/L)	TN (mg/L)	TP (mg/L)
1.5	24.66	302.22	18.56	33.40	4.28
2.5	1261.02	745.37	47.01	85.45	9.88
7.0	740.17	1219.39	74.86	135.66	16.53
14.0	520.92	71.90	7.45	14.12	0.44
13.5	219.18	168.88	3.15	7.03	1.32
16.0	520.92	144.33	5.62	11.20	0.22
32.5	400.00	50.00	8.00	15.00	0.50

**Table 5:** Flow and concentrations of non-point sources.

Number	Inflow Range (km)	Flow (m^3^/d)	COD (mg/L)	NH_3_-N (mg/L)	TN (mg/L)	TP (mg/L)
1	0~5	18815.34	263.63	46.44	130.02	8.09
2	0~6	5924.38	282.55	30.03	84.07	7.20
3	0~4.5	3465.21	329.94	36.98	103.54	6.21
4	5~15	4441.64	304.84	33.35	93.39	7.28
5	5~17	7117.81	271.48	32.30	90.44	6.50
6	28~35	8400.00	262.97	31.82	89.11	6.84
7	22~29	1900.27	423.94	39.30	110.03	6.62
8	15~23	3626.30	324.86	27.37	76.65	7.36
9	31~35	1873.97	426.53	55.37	155.03	6.48

#### 2.3.2. Parameters

The extent of parameters (Table 6) that QUAL2K demanded were determined from a large number of studies including documentation for the stream water quality model QUAL2E [26], the QUAL2K user manual [27] and the Environment Protection Agency guidance document [28]. The model was validated with data of 2011 using parameters that were calibrated with data of 2010. The calibrated parameters are shown in Table 6. The remaining parameters were set by the default values in the model.

**Table 6:** Calibrated parameters for the Wujin River water quality modeling.

Parameter	Value	Units	Symbol	Range
O_2_ reaeration model	Internal			
Fast CBOD oxidation rate	0.23	day^−1^	*k_dc_*	0.02–4.2
Organic N hydrolysis	0.27	day^−1^	*k_hn_*	0–5
Organic N settling velocity	0.05	m/day	*v_on_*	0–2
Ammonium nitrification	0.29	day^−1^	*k_na_*	0–10
Nitrate denitrification	0.24	day^−1^	*k_dn_*	0–2
Organic P hydrolysis	0.46	day^−1^	*k_hp_*	0–5
Organic P settling velocity	1.0	m/day	*v_op_*	0–2
Inorganic P settling velocity	0.59	m/day	*k_ip_*	0–2
Bottom algae				
Growth model	zero-order			
Max Growth rate	60	mgA/m^2^/day	*C_gb_*	0–500
Respiration rate	0.25	day^−1^	*k_rb_*	0.05–0.5
Excretion rate	0.5	day^−1^	*k_eb_*	0–0.5
Death rate	0.25	day^−1^	*k_db_*	0–0.5

#### 2.3.3. Implementation of the Model

The QUAL2K model can follow the specific circumstances of users to set the parameter values and transform the simulation equation to satisfy the user requirements. In this study, the parameters of *k_hc_, k_dn_, k_dt_* (Detritus Dissolution rate) were set to 0 and *F_oxc_* (CBOD_f_ attenuation due to low oxygen) was set to 1, so CBOD_f_ represents the concentration of COD. The *k_dc_* was then set as the COD comprehensive degradation coefficient; thus, QUAL2K can be used to simulate the changes in COD [27,29]. The calculation time step was set to 5.6 min to ensure the model was maintained in the steady-state. The integration solution was handled with Euler’s method [29]. The model was validated with another different data, which was set without altering the calibrated parameters, so that the calibrated model can forecast the component concentration accurately.

### 2.4. Evaluation Method of Load Reduction Scenarios

#### 2.4.1. Analytic Hierarchy Process

Pollution load reduction scenarios were compared using an analytic hierarchy process to comprehensively evaluate their effects for various evaluation indicators [3]. To determine the relative importance of these evaluation indicators, their respective weight coefficients were obtained using Saaty’s ratio-scale method [30,31]. First, initial weight coefficients and normalized weight coefficients for each of the evaluation indicators were calculated; second, the comprehensive effects of each scenario were calculated.

#### 2.4.2. The Delphi Method

According to the actual situation of the study area, qualitative forecasting methods named the Delphi method was used to predict the increasing rates of evaluation indicators. The Delphi method was originally developed in the 1950s and first used in 1964 by the Rand Corporation in Santa Monica, California [32,33]. The accuracy of the forecast results to a large extent dependent on the breadth, depth and experience of the expert knowledge, so the choice of experts is very important [34]. During the last ten years, the Delphi method was used more often especially for natural science and technology field [35,36,37,38].

## 3. Results and Discussion

### 3.1. Calibration and Validation

**Figure 3:**
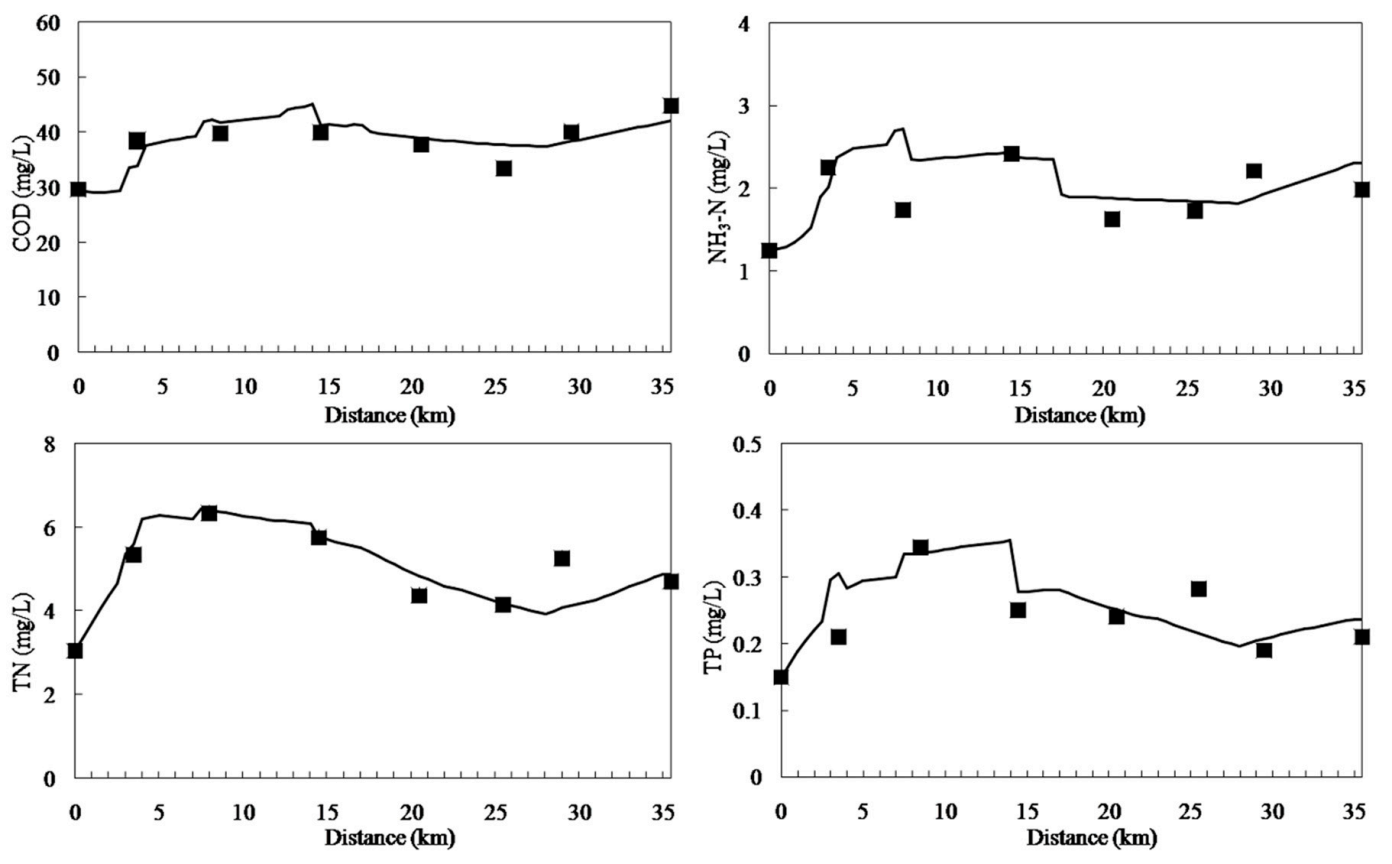
Water quality calibration results for the Wujin River.

**Figure 4:**
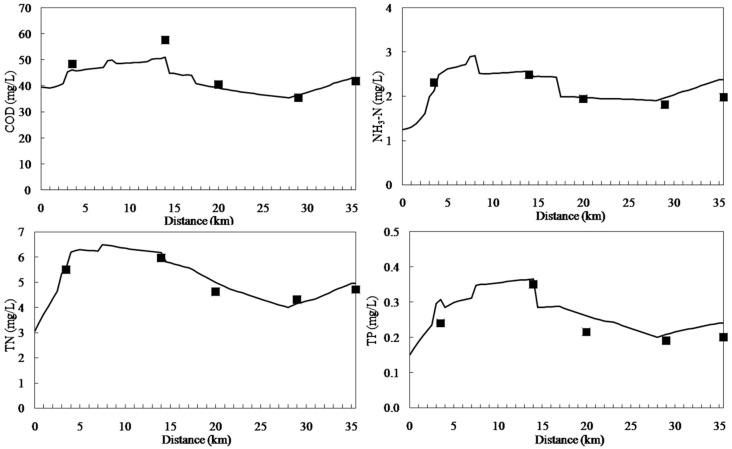
Water quality validation results for the Wujin River.

The model was calibrated with data of 2010 and validated with data of 2011. Figure 3 and Figure 4 display the results of calibration and validation, respectively. As shown in Figure 3, water quality improved from the headwater to the downstream areas. The concentrations of pollutants increased at 0–5 km downstream because of the afflux of a large number of point source and non-point source pollutants and because Cailing River has high concentrations of pollutants. The concentrations of COD, NH_3_-N, TN, and TP reduced suddenly at 14 km downstream because of large quantities of water with low concentrations of pollutants from the Xilicao River tributary flowing into Wujin River. In addition, the concentrations of various water quality factors increased at 28 km downstream because of non-point source pollutants discharged. The calibration results of the qual2k model were in accordance with the monitoring values. The validation results (Figure 4) were very good indicated that the calibrated parameters are very reliable.

### 3.2. Calculation of Pollution Load

We conducted a pollution load investigation and analysis of the Wujin River watershed. Specifically, we studied the states of water environmental quality, the emissions of industrial wastewater, domestic sewage, and agricultural non-point sources. Based on the results, average annual emissions were 6020.10 t, 675.99 t, 1815.47 t, and 165.90 t for COD, NH_3_-N, TN, and TP, respectively. Table 7 shows that domestic sewage emissions accounted for a high percentage of COD, NH_3_-N, TN, and TP emissions.

**Table 7:** Annual emissions of COD, NH3-N, TN, and TP in Wujin River watershed.

Pollution Sources	COD(t/a)	NH_3_-N(t/a)	TN(t/a)	TP(t/a)
Industrial wastewater	983.08	31.76	89.10	6.47
Domestic sewage	4069.70	533.20	1492.40	105.32
Agricultural non-point sources	724.90	103.20	212.00	52.50
Total	6020.10	675.99	1815.47	165.90

### 3.3. Simulation and Evaluation of Pollution Load Reduction Scenarios

#### 3.3.1. Design of Pollution Load Reduction Scenarios

According to the water environment status and control objectives of the Wujin River watershed, a variety of pollution load reduction scenarios were designed including: (1) Control the pollution load from point sources and non-point sources simultaneously; (2) Control only point sources; (3) Control only non-point sources; (4) Control only the rivers using ecological purification technologies; (5) Control point sources, non-point sources, and the rivers simultaneously.

#### 3.3.2. Simulation of Pollution Load Reduction Scenarios

Water quality objectives must be determined based on water environmental management requirements; according to the requirements of Jiangsu Province, the water quality objectives of the Wujin River watershed are Grade IV water quality standards [25]. In this study, the end of the river was used for water quality control. Grade IV water quality standards are 30.0 mg/L, 1.5 mg/L, 1.5 mg/L, and 0.3 mg/L for COD, NH_3_-N, TN and TP, respectively. By simulating different scenarios, the load reduction rate of different scenarios was obtained such that the water quality at the end of Wujin River reached the required standards. The initial concentrations of various programs are Grade IV water quality standards. In the simulation, the input pollution concentrations of COD, NH_3_-N, TN and TP were adjusted by trial and error until the water quality simulation results met the water quality objectives.

**Figure 5:**
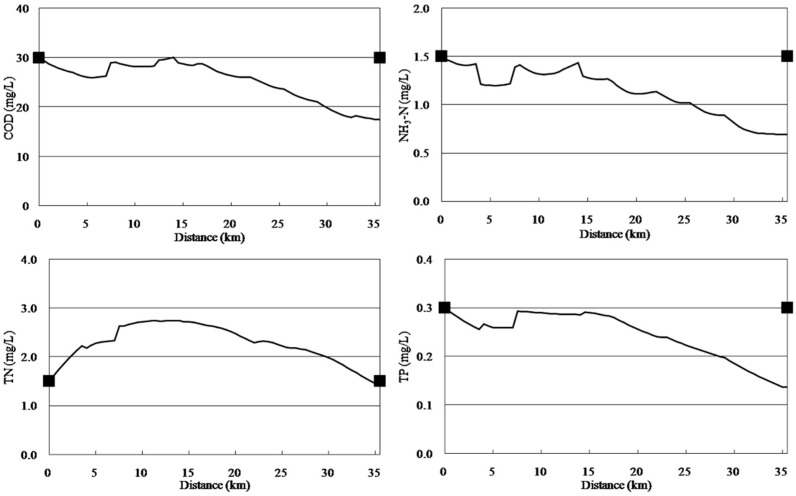
Simulation curves from implementing point source and non-point source simultaneous reductions of 58.77%.

**Point sources and non-point sources simultaneously reduced.** Simultaneous point source and non-point source reductions of 13.35%, 27.26% and 37.08% for COD, NH_3_-N, and TP were required, respectively, for concentrations to meet the standards at the end of the river. As shown in Figure 5, simultaneous point source and non-point source reductions of 58.77% were required for TN concentrations to meet the standards, meanwhile, COD, NH_3_-N, and TP concentrations were lower than the Grade IV standards; therefore, the water quality of the river would meet the required standards.

**Figure 6:**
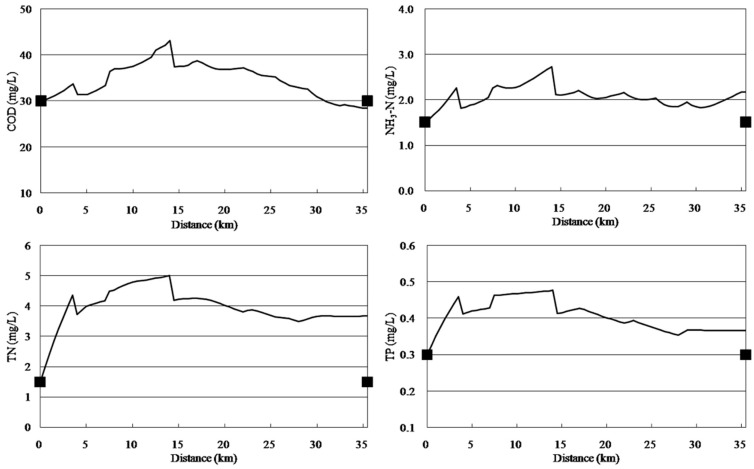
Simulation curves from implementing point source reduction of 100%.

**Point source reduction.** The simulation results for the point source reduction scenario are shown in Figure 6. The results show that the pollution load of point sources needed to be reduced by 83.41% for COD concentrations to meet the standards; however, NH_3_-N, TN, and TP did not meet the standards with a 100% point source pollution load reduction. This is because non-point source pollutants (including domestic sewage and agricultural non-point source pollutants) in the watershed accounted for more than 80% of pollutant emissions; therefore, reducing only the point source pollution load did not achieve the water quality standards.

**Figure 7:**
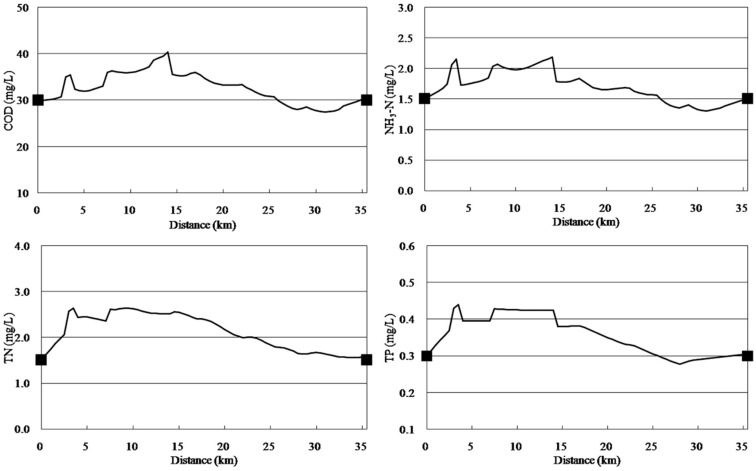
Simulation curves from implementing non-point source reduction, as required for the concentrations of various water quality variables to meet the standards.

**Non-point source reduction.** The simulation results for the non-point source reduction scenario are shown in Figure 7. The results show that pollution load of only non-point sources needed to be reduced by 17.14%, 29.73%, 40.06% and 62.59% for COD, NH_3_-N, TP and TN concentrations, respectively, to meet the standards.

**Control of** **rivers using ecological purification technologies.** The scenario based on implementation of only water pollution control technologies and ecological purification measures in the river to control and govern the pollution of the river network resulted in an increase in the water quality degradation coefficient of Wujin River. This would improve the degradation and absorption properties of pollutants such as nitrogen and phosphorus, so the river water quality can be improved.

**Figure 8:**
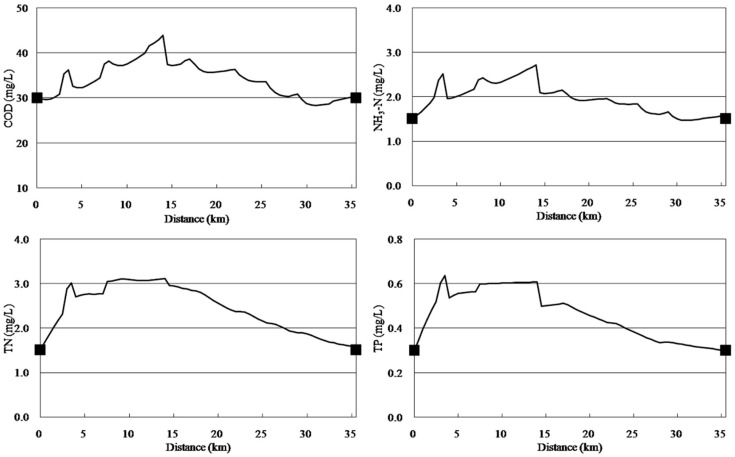
Simulation curves from implementing the river control measures.

As shown in Figure 8, river pollution control and ecological purification measures resulted in degradation coefficients of 0.38 d^−1^, 0.52 d^−1^, 0.41 d^−1^, 0.59 d^−1^, 0.53 d^−1^, and 0.65 d^−1^ for COD, organic nitrogen, ammonia nitrogen, nitrate nitrogen, organic phosphorus and inorganic phosphorus, respectively, and COD, NH_3_-N, TN, and TP concentrations would reach the standards at the river’s end. However, the biomass, aquatic plant density and size of project required to achieve these parameters needs further site-based experimental studies [3,39].

**Control point sources, non-point sources, and the rivers simultaneously.** The pollution loads of COD, NH_3_-N, TN, and TP were all reduced by about 30% as a result of river pollution control projects [40,41,42,43]. Therefore, this scenario assumed that river control can reduce 30% of the pollution load; hence, the pollution load of point sources and non-point sources was reduced on this basis.

**Figure 9:**
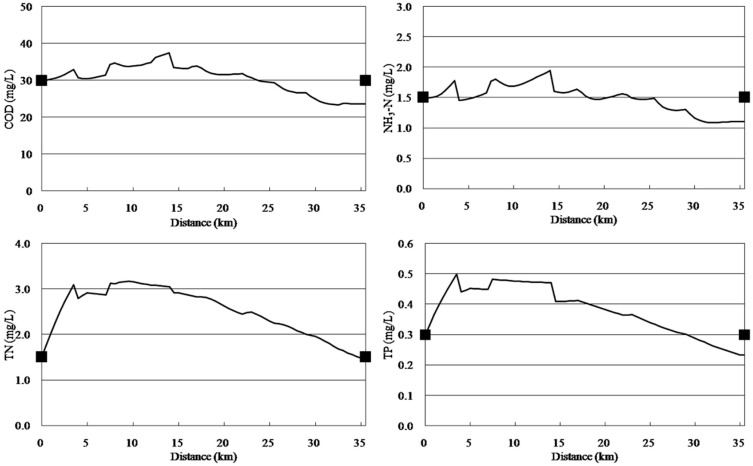
Simulation curves from implementation of river control measures and a pollution load reduction of 23.20%.

The simulation results showed that COD, NH_3_-N, and TP concentrations met the standards without reducing the pollution load of point sources and non-point sources; however, point source and non-point source pollution load reductions of 23.20% are needed for TN concentrations to meet the standards (Figure 9). The results show that the implementation of pollution control engineering and ecological purification measures in the river results in COD, NH_3_-N, and TP concentrations that meet the standards. Integrated pollution control measures or environmental management countermeasures need to be implemented to reduce the discharge of pollution load by 23.20% and, combined with the implementation of ecological purification measures in the rivers, would result in the water quality at the end of the river meeting the standards.

Based on the above discussion, the simulation results of various load reduction scenarios are shown in Table 8.

**Table 8:** Simulation results for various load reduction scenarios.

Type of Scenario	Objective	Reduction Rate of Point Sources	Reduction Rate of Non-Point Sources
Control point sources and non-point sources	COD up to standard	13.35%	13.35%
	NH_3_-N up to standard	27.26%	27.26%
	TP up to standard	37.08%	37.08%
	TN up to standard	58.77%	58.77%
Control point sources	COD up to standard	83.41%	—
	NH_3_-N up to standard	100%, still not up to standard	—
	TP up to standard	100%, still not up to standard	—
	TN up to standard	100%, still not up to standard	—
Control non-point sources	COD up to standard	—	17.14%
	NH_3_-N up to standard	—	29.73%
	TP up to standard	—	40.06%
	TN up to standard	—	62.59%
Control the rivers	water quality up to standards	—	—
Control point sources, non-point sources, and rivers	water quality up to standards	23.20%	23.20%

#### 3.3.3. Evaluation of Pollution Load Reduction Scenarios

The following four pollution load reduction scenarios were selected for further evaluation because in these scenarios COD, NH_3_-N, TN, and TP concentrations each met the standards at the river’s end.

Scenario A: The pollution loads of point sources and non-point sources were simultaneously reduced by 58.77%. Scenario B: The pollution loads of non-point sources were reduced by 62.59%. Scenario C: Implementation of river pollution control and ecological purification measures resulted in river degradation coefficients of 0.38 d^−1^, 0.52 d^−1^, 0.41 d^−1^, 0.59 d^−1^, 0.53 d^−1^, and 0.65 d^−1^ for COD, organic nitrogen, ammonia nitrogen, nitrate nitrogen, organic phosphorus, and inorganic phosphorus, respectively. Scenario D: The pollution loads of point sources and non-point sources were reduced by 23.20% based on a 30% pollution load reduction for river control.

An evaluation indicator system for the pollution load reduction scenarios was constructed using the evaluation indicators of environmental, social, economic, ecological and investment (Table 9). Table 10 shows a judgment optimization matrix for pairwise comparisons of the five evaluation indicators using the ratio-scale method [44,45,46], these data were obtained based on social and economic development, people's environmental expectations and a field survey of the watershed.

**Table 9:** The evaluation indicator system of pollution load reduction scenarios.

Indicators	Factor	Correlation
Environmental	COD; NH_3_-N; TN; TP	Positive
Social	Population; Scientific and cultural quality; Environmental awareness	Positive
Economic	Gross National Product; Per capita income	Positive
Ecological	Living environment; Per capita water resources; Vegetation cover	Positive
Investment	The proportion of investment accounting for Gross National Product; Investment	Negative

**Table 10:** Judgment optimization matrix for pairwise comparisons of the evaluation indicators.

Indicators	Environmental	Social	Economic	Ecological	Investment
Environmental	1	4/5	3/2	5/4	2
Social	5/4	1	2	5/3	3
Economic	2/3	1/2	1	3/4	4/3
Ecological	4/5	3/5	4/3	1	3/2
Investment	1/2	1/3	3/4	2/3	1

Initial weight coefficients and normalized weight coefficients (Table 11) for each of the evaluation indicators were calculated. For each of the pollution load reduction scenarios, the increasing rates of the five evaluation indicators were forecast based on the Delphi method [47,48,49] (Table 12). The comprehensive evaluation values for the effects of each of the four load reduction scenarios were calculated using the weight coefficients of the evaluation indicators in Table 11 and the increasing rates of the evaluation indicators in Table 12. The comprehensive evaluation values for the scenarios A, B, C, and D were 0.246, 0.217, 0.180, and 0.194, respectively.

**Table 11:** Weight coefficients of the evaluation indicators.

Weight Coefficients	Environmental	Social	Economic	Ecological	Investment
Initial weight coefficient (W_i_')	1.24	1.61	0.80	0.99	0.61
Normalized weight coefficient (W_i_)	0.23	0.30	0.16	0.19	0.12

**Table 12:** Evaluation indicator increasing rates of each load reduction scenarios.

Indicators Increasing Rates	Scenario A	Scenario B	Scenario C	Scenario D
Environmental	42%	35%	26%	31%
Social	40%	35%	25%	30%
Economic	−13%	−8%	5%	−3%
Ecological	36%	30%	21%	25%
Investment	15%	11%	3%	8%

The comprehensive evaluation values indicate that the scenarios in order of decreasing effect were A, B, D, and C. The comprehensive evaluation effect of Scenario A was the largest, suggesting that control the discharge of point sources and non-point sources from the sources is the optimal measure for prevention and control of pollution. This scenario had positive environmental, social and ecological effects; however, it would affect economic development and requires large investment. The comprehensive evaluation effect of Scenario C was the smallest, indicating that not controlling from the sources but controlling after pollution is an ineffective means of environmental pollution control; however, economic growth was ensured. The comprehensive evaluation effect of Scenario D, which controlled the sources as well as the environmental pollution, was between that of A and C; this result is reasonable and logical.

Table 7 shows that domestic sewage emissions accounted for 67.60%, 78.89%, 82.20% and 63.48% for COD, NH_3_-N, TN, and TP, respectively, of total emissions of pollution load. The pollutants discharged in domestic sewage can be removed using measures such as home sewage processing purification tanks, home-constructed wetland systems, and centralized sewage treatment plants. The average removal of home wetland system treatments is 93.0%, 88.4%, 87.7%, 97.0% and 89.6% for COD, ammonia nitrogen, total phosphorus, total suspended solids and turbidity, respectively [50]. The removal of home sewage distributed processing purification tanks is 93%, 76% and 91% for COD, TN, and TP, respectively [51,52]. Therefore, COD, ammonia nitrogen, TN and TP of domestic sewage can be removed by more than 80% using these measures; thus, pollution load reductions of 54.08%, 63.11%, 65.80% and 50.78% for COD, ammonia nitrogen, TN, and TP, respectively, can be achieved in the Wujin River watershed. According to the pollution load reduction rates shown in Table 8, the COD, ammonia nitrogen, TN, and TP reductions meet the standards at the river’s end. Based on the above, Scenarios A and B have the greatest feasibility and operability.

## 4. Conclusions

This study aimed to establish the response relationship between the pollution load and water quality of the watershed. A variety of pollution load reduction scenarios were simulated using the calibrated QUAL2K model. As the evaluation results showed, controlling the emission of pollutants from the sources is the optimal measure for pollution prevention and control. The method of “Treatment after Pollution” has bad comprehensive effects for the environment, society and ecology; however, it had positive economic growth effects. Different load reduction scenarios have different effects; hence, environmental managers can select and implement different scenarios depending on particular objectives. The QUAL2K model proved to be an effective tool for simulation and evaluation of river pollution load reduction scenarios. A method was produced for pollution control, assisting environmental management decision-making on selection of suitable pollution load reduction scenarios, and for forecasting the effects of these scenarios. The results of the study provide a basis for choosing appropriate pollution load reduction scenarios for water environmental management including implementation of water quality improvement measures.
